# Supplementary material for: MCTS2 and distinct eIF2D roles in uORF-dependent translation regulation revealed by in vitro re-initiation assays
Source: EMBO J. 2025 Jan 2;44(3):854–76. doi: 10.1038/s44318-024-00347-3 (PMC11790910; doi:10.1038/s44318-024-00347-3)
Supplement: Supplementary file 1 — Appendix [file 44318_2024_347_MOESM1_ESM.pdf]

MCTS2 and distinct eIF2D roles in uORF-dependent translation regulation revealed by *in vitro* re-initiation assays

Romane Meurs[1], Mara De Matos[1], Adrian Bothe[2], Nicolas Guex[3], Tobias Weber[4], Aurelio A. Teleman[4], Nenad Ban[2], David Gatfield[1*]

Appendix

Affiliations:

[1] Center for Integrative Genomics, University of Lausanne, 1015 Lausanne, Switzerland.

[2] Department of Biology, Institute of Molecular Biology and Biophysics, ETH Zurich, 8093 Zurich, Switzerland.

[3] Bioinformatics Competence Center, University of Lausanne, 1015 Lausanne, Switzerland.

[4] Division of Signal Transduction in Cancer and Metabolism, German Cancer Research Center (DKFZ), Heidelberg, Germany.

[*] Corresponding author

Email addresses: romane.meurs@unil.ch, mara.dematos@unil.ch, adrian.bothe@mol.biol.ethz.ch, nicolas.guex@unil.ch, tobias.weber@dkfz-heidelberg.de, a.teleman@dkfz-heidelberg.de, ban@mol.biol.ethz.ch, david.gatfield@unil.ch

Table of contents

Appendix Figure S1 ………………………………………. page 3

Appendix Figure S1 - Legend ……………………………… page 3

Appendix Figure S2 ………………………………………. page 4

Appendix Figure S2 - Legend ……………………………… page 5

Appendix Figure S3 ………………………………………. page 6

Appendix Figure S3 - Legend ……………………………… page 6

Appendix Figure S4 ………………………………………. page 7

Appendix Figure S4 - Legend ……………………………… page 7

Appendix Figure S5 ………………………………………. page 8

Appendix Figure S5 - Legend ……………………………… page 8

Appendix Figure S6 ………………………………………. page 10

Appendix Figure S6 - Legend ……………………………… page 10

Appendix Figure S7 ………………………………………. page 11

Appendix Figure S7 - Legend ……………………………… page 11

Appendix Figure S8 ………………………………………. page 12

Appendix Figure S8 - Legend ……………………………… page 12

A

mouse *Klhdc8a* 5’ UTR and beginning of CDS (ENSMUST00000046071)

```
  1 GTCTCCGACCCTGTAGACACTGCAGGCCCTGCCCTCGCTCGCCCTGGTCGCCCGGGGCTG    60
 61 ACGCACAGGCCACAGCCTCCGCCTCGGGGCGCGTGGGCTCCTGCCCAGGTGTGGAAGCTG   120
121 CGGGGAGGCGGTGCGGGGAACCCGCGTCCTCCGGCCGGAGCTCGGCTTCTGGCGACCGCT   180
181 CTCCTGCGCGGGTGACGGCGGCGTGCGGGCTGGGAAGGGCAGGGAGCGCTGTCCTCCGCT   240
241 CCGCGGCTGCCAGGCGGACCGTGAAAGCTTCGCGCGGCTGTAGGCGCCGCCCGGGAGCTA   300
301 GTGCTGGGCGAGCGCGGCGCCCACGCGCGGTGGGGTGGGCTCCGAGCTCCGGGGGACCGG   360
361 GCGGGGGCGGGGCGCATCCTGCAAAGTTGACCAAGGGTGAGCTACCGCCGGCGCCCTGCT   420
421 GCCCTAGATTCGCCAGGTTCTTTCCGCGGAGCCAGGCGTGGACGTCGCTGCCCCGCTGAC   480
481 ACCCAAGGGACCCGGAGAAGCCAGCGGGCGTCGGCAGGGAGCCCGTGCAGCGCGAGATGG   540
                                                     uORF -M--
541 AGGCTGAGAGAGGGGACGCGCCGACCGGCGCCGTGTAGCCCGCGGGCCCCGGGGCTGGAC   600
    E--A--E--R--G--D--A--P--T--G--A--V--*-
601 CTTGCCCGGACGCTGTGGGGGCGCTCGAGCAGGGCCCTCCCCCGGGCTGCCATGGAAGTG   660
                                                CDS -M--E--V-
661 CCCAATGTCAAGGACTTTCAGTGGAAGCGCCTTGCTCCACTGCCTAGCCGCCGGGTCTAT   720
    -P--N--V--K--D--F--Q--W--K--R--L--A--P--L--P--S--R--R--V--Y-
```

B

mouse *Asb8* 5’ UTR and beginning of CDS (ENSMUST00000143400)

```
  1 AGAATATCATGTCGCTCAGAGGCTCTGACCATGCAGCACCCAGAGAGCACAAGGGGTCTG    60
 61 GGCCAAGAGGGAGGGGAATTTCCTGGAGAAGCACTGAAGATCATGGGAAGCAGGTGCAGA   120
121 GGCCCAGGAGCGTGGCTGATCCAGTCCAATTCCAGGCAGTTTGGAGCATGTGAACACACT   180
                                         uORF -M--*-
181 CTGAGCCTTGATGAGTTCCAGCATGTGGTATATTATGCAGAGCATTCAGAGCAAATACTC   240
         CDS -M--S--S--S--M--W--Y--I--M--Q--S--I--Q--S--K--Y--S
```

C

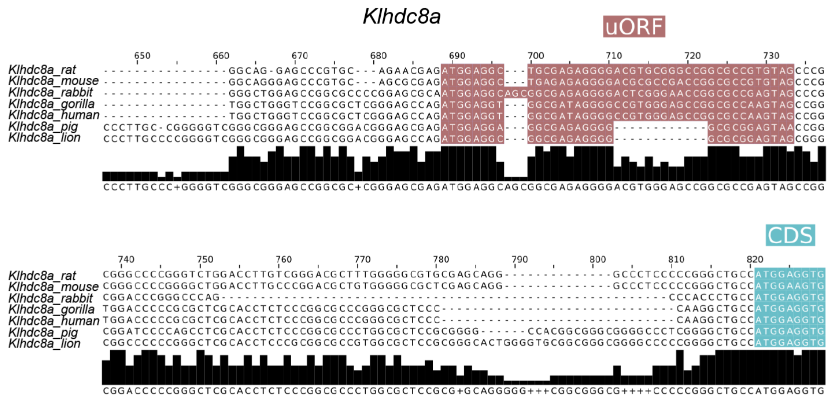

D

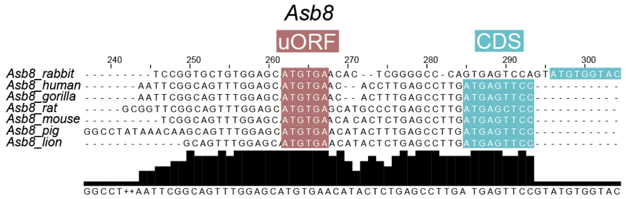

Appendix Figure S1. *Klhdc8a* and *Asb8* both contain a single AUG uORF that is conserved across mammals. (A) Nucleotide sequences of mouse *Klhdc8a* 5′ UTR and beginning of CDS. Position and amino acid sequence of uORF and CDS are shown and highlighted in colours. **(B)** As in (A), but for *Asb8* 5′ UTR and beginning of CDS. **(C)** Sequence alignments of the 3′ portion of the 5′ UTR of *Klhdc8a* from rat, mouse, rabbit, gorilla, human, pig and lion show conservation of their uORF sequences. **(D)** As in (C) for *Asb8*.

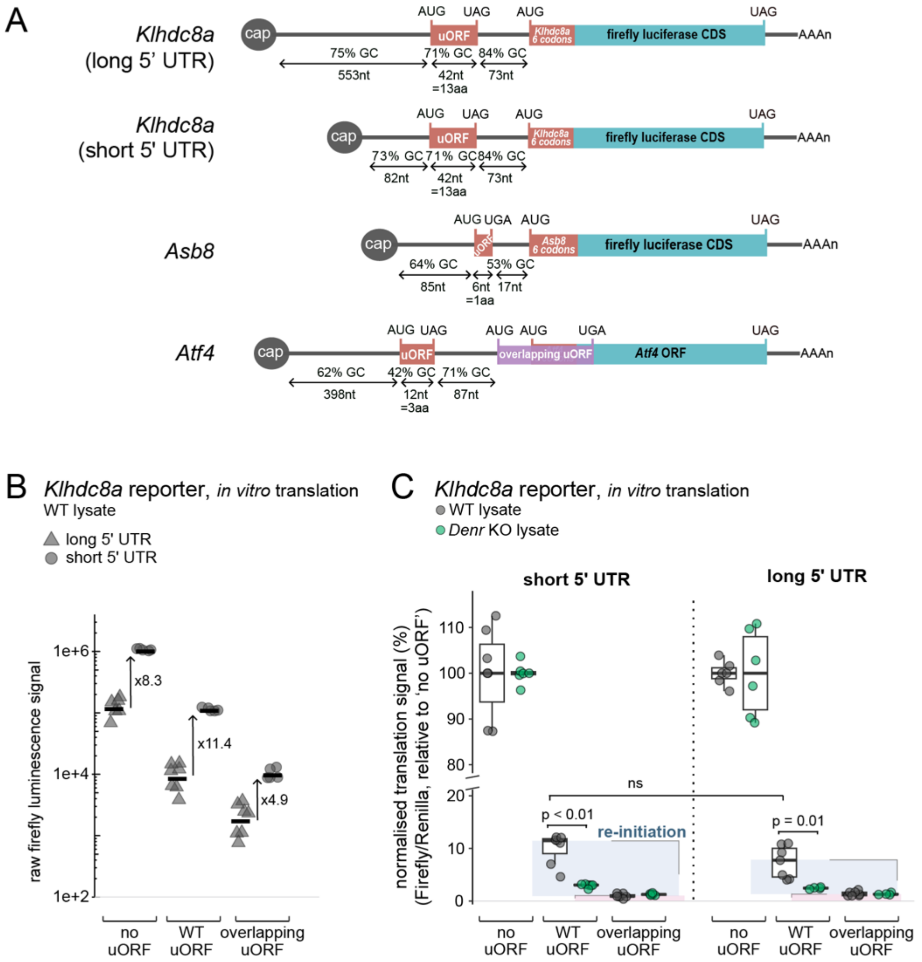

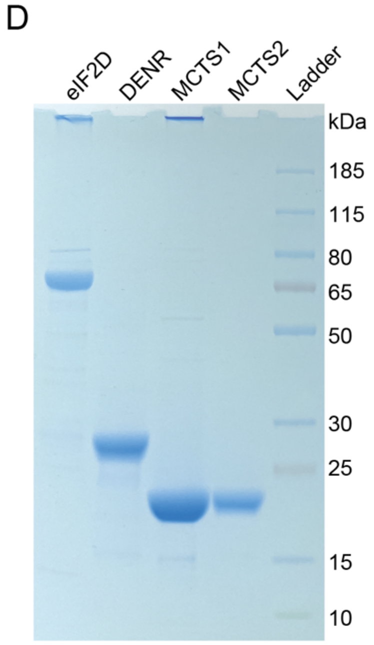

E

eIF2D:

MFAKAFRVKSNTAIKGSDRRKLRADVTTAFPTLGTDQVSELVPGKEELNI
VKLYAHKGDAVTVYVSGGNPILFELEKNLYPTVYTLWSYPDLLPTFTTWP
LVLEKLVGGADLMLPGLVMPPAGLPQVQKGDLCAISLVGNRAPVAIGVAA
MSTAEMLTSGLKGRGFSVLHTYQDHLWRSGNKSSPPSIAPLALDSADLSE
EKGSVQMDSTLQGDMRHMTLEGEEENGEVHQAREDKSLSEAPEDTSTRGL
NQDSTDSKTLQEQMDELLQQCFLHALKCRVKKADLPLLTSTFLGSHMFSC
CPEGRQLDIKKSSYKKLSKFLQQMQQEQIIQVKELSKGVESIVAVDWKHP
RITSFVIPEPSPTSQTIQEGSREQPYHPPDIKPLYCVPASMTLLFQESGH
KKGSFLEGSEVRTIVINYAKKNDLVDADNKNLVRLDPILCDCILEKNEQH
TVMKLPWDSLLTRCLEKLQPAYQVTLPGQEPIVKKGRICPIDITLAQRAS
NKKVTVVRNLEAYGLDPYSVAAILQQRCQASTTVNPAPGAKDSLQVQIQG
NQVHHLGWLLLEEYQLPRKHIQGLEKALKPGKKKENLYFQ

DENR:

SNAMAADISESSGADCKGDPRNSAKLDADYPLRVLYCGVCSLPTEYCEYM
PDVAKCRQWLEKNFPNEFAKLTVENSPKQEAGISEGQGTAGEEEEKKKQK
RGGRGQIKQKKKTVPQKVTIAKIPRAKKKYVTRVCGLATFEIDLKEAQRF
FAQKFSCGASVTGEDEIIIQGDFTDDIIDVIQEKWPEVDDDSIEDLGEVK
K

MCTS1:

MFKKFDEKENVSNCIQLKTSVIKGIKNQLIEQFPGIEPWLNQIMPKKDPV
KIVRCHEHIEILTVNGELLFFRQREGPFYPTLRLLHKYPFILPHQQVDKG
AIKFVLSGANIMCPGLTSPGAKLYPAAVDTIVAIMAEGKQHALCVGVMKM
SAEDIEKVNKGIGIENIHYLNDGLWHMKTYKENLYFQ

MCTS2:

MFKKFDEKESVSNCIQLKTSVIKGIKSQLVEQFPGIEPWLNQIMPKKDPV
KIVRCHEHTEILTVSGELLFFRQRKGPFCPTLRLLHKYPFILPHQQVDKG
AIKFVLSGANIMCPGLTSPGAKLYPAAVDTIVAVTAEGKQHALCVGVMKM
SAEDIEKVNKGIGIENIHYLNDGLWHMKTYKENLYFQ

Appendix Figure S2. Shortening of *Klhdc8a* 5′ UTR increases the raw luciferase signal without affecting re-initiation rate. (A) Schematic representation of the short and long 5′ UTR of *Klhdc8a* and the whole 5′ UTR of *Asb8* WT reporters used in this study compared to the mouse *Atf4* transcript. uORF lengths, upstream and downstream sequence lengths and GC contents are indicated. **(B)** Raw luminescence signal of long vs. short 5′ UTRs of *Klhdc8a* reporters after *in vitro* translation in HeLa WT lysate shows higher signal upon shortening of the 5′ UTR. **(C)** Normalised luminescence signal (firefly/Renilla) of the *Klhdc8a* reporters after *in vitro* translation in WT and *Denr* KO HeLa lysates of the short 5′ UTR reporters (left) and the long 5′ UTR reporters (right); significance calculated using two-tailed unpaired *t*-test. **(D)** Coomassie-stained SDS-PAGE gel with 1 µg each of purified recombinant proteins (eIF2D, DENR, MCTS1, MCTS2, as indicated) validates the purity of the prepared proteins. **(E)** Sequences of the recombinant proteins after TEV cleavage. Underlined: additional amino acids due to the expressed construct/tag remnants that are not encoded by the genomic sequence.

Quantifications of re-initiation rates in *in vitro* assays

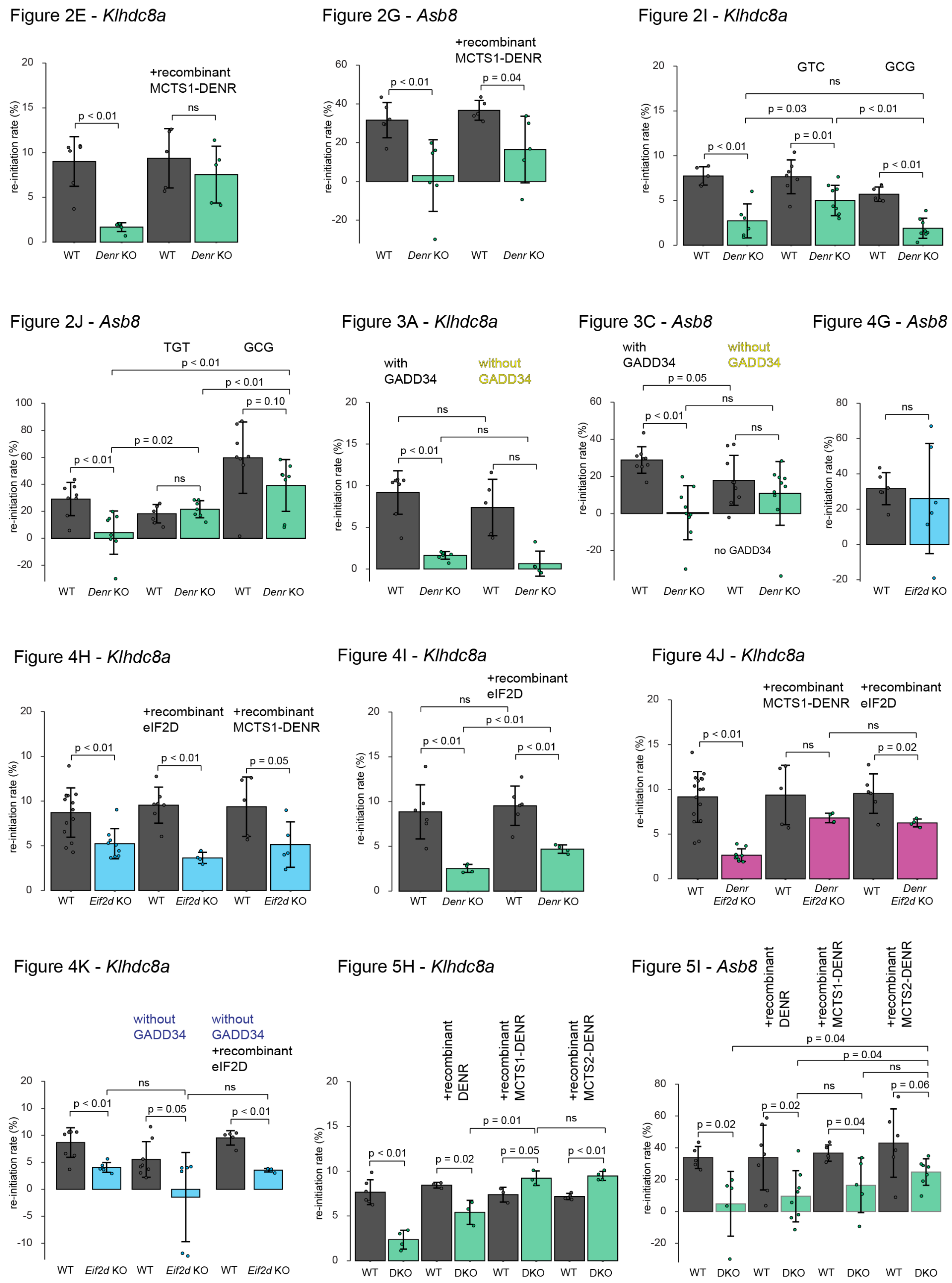

Appendix Figure S3. Quantification of re-initiation rates from *in vitro* assays. Plotting of calculated re-initiation values (represented by the blue shaded boxes in the main figures) across all *in vitro* assays from the manuscript main figures, as indicated. Significance calculated using two-tailed unpaired *t*-test.

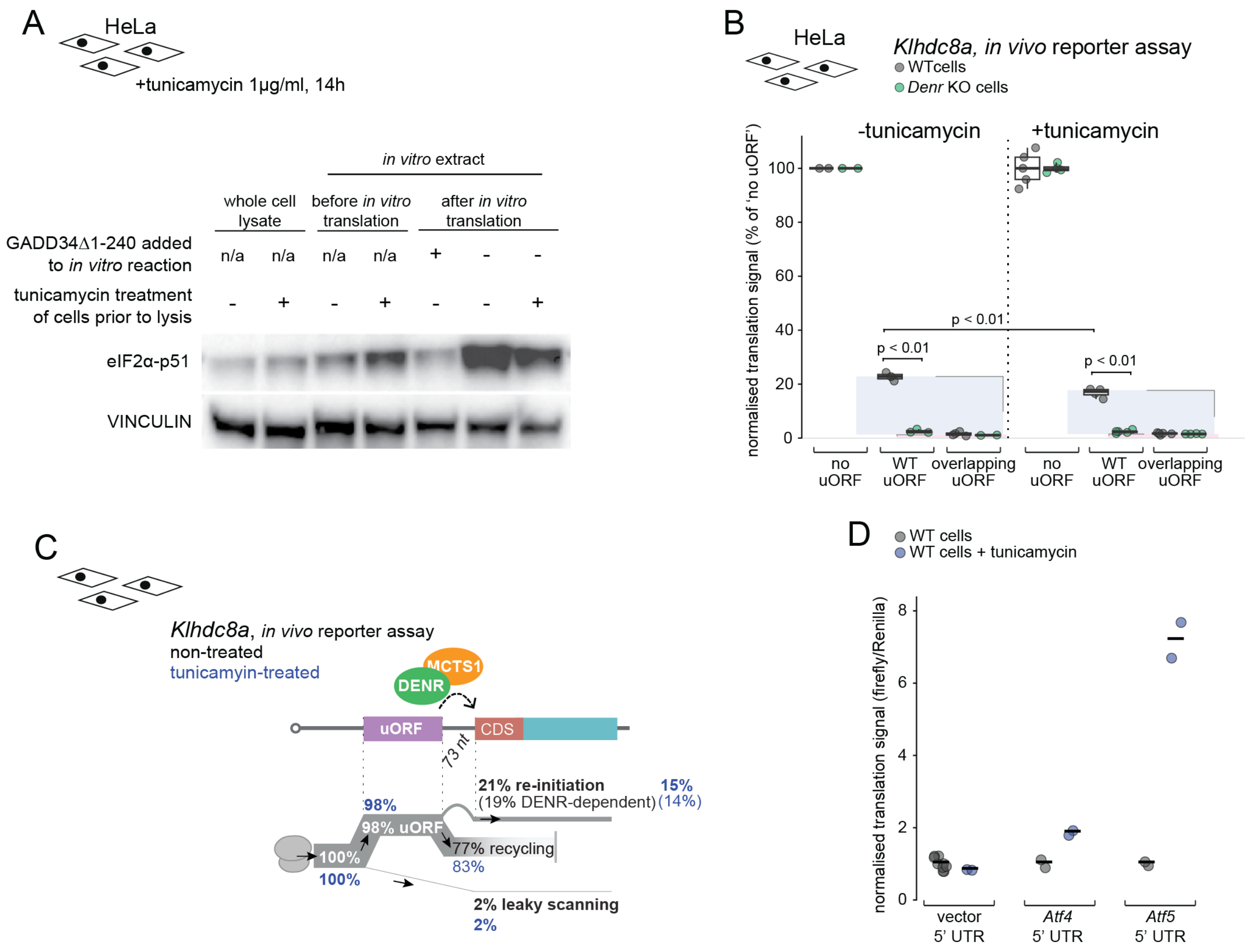

Appendix Figure S4. Effect of tunicamycin treatment on *Klhdc8a* reporter *in vivo*.

(A) Western blot analysis of whole cell protein extracts or translation-competent extracts before and after *in vitro* translation, non-treated or treated with 1 µg/ml tunicamycin before cell harvest or with 16 ng/µl GADD34Δ1-240 during *in vitro* translation, reveals that the eIF2α-p51 phosphorylation level increase is stronger in *in vitro* translation reactions without GADD34Δ1-240 than after tunicamycin-mediated ISR induction *in vivo*. **(B)** Normalised luminescence signal (firefly/Renilla) of *Klhdc8a* reporters after transduction in WT and *Denr* KO cells non-treated (left) and treated with tunicamycin (right). **(C)** Schematic representation of ribosomal fluxes on *Klhdc8a* 5′ UTR estimated from the results shown in panel (B). Values measured in non-treated cells are shown in black and those quantified from tunicamycin-treated cells in blue. **(D)** Normalised translation signal (firefly/Renilla) of *Atf4* and *Atf5* reporters measured in transduced HeLa WT and tunicamycin-treated cells confirms the increased translation of the two reporters upon eIF2α phosphorylation.

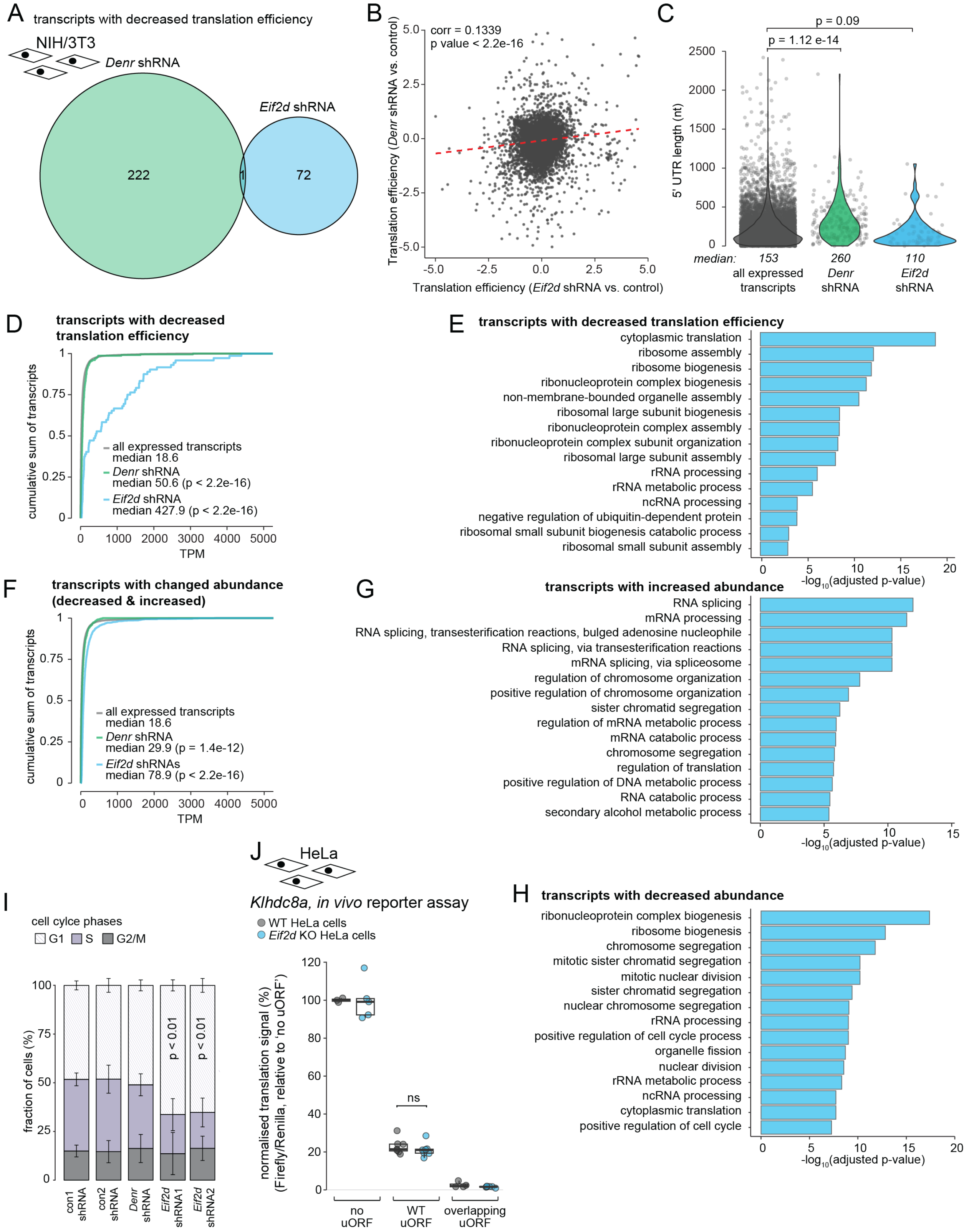

Appendix Figure S5. Ribosome profiling and *in vivo* dual luciferase assay reveals differential effects of *Denr* and *Eif2d* knock-down. (A) Venn diagram of transcripts with reduced TE upon *Denr* or *Eif2d* knock-down shows poor overlap. **(B)** Correlation analysis between translation efficiency in *Denr* and *Eif2d* knock-down cells shows a weak but significant correlation (Pearson correlation = 0.1339, p-value < 2.2e-16). **(C)**

Violin plot of 5′ UTR lengths of all expressed transcripts (n = 9203, grey) vs. transcripts with lower TE upon *Denr* depletion (n = 221, green) and *Eif2d* depletion (n = 73, blue). *Eif2d*-responsive genes tend to have shorter 5′ UTRs (median = 110 nt) than overall expressed transcripts (median = 153 nt) (p = 0.09, Kolmogorov–Smirnov test). **(D)** Cumulative distributions of transcript per kilobase million (TPM) of all expressed transcripts, transcripts with decreased TE upon *Eif2d* knock-down and transcripts with decreased TE upon *Denr* knock-down show a strong enrichment for highly abundant transcripts among *Eif2d*-responsive mRNAs (p-value calculated using Kolmogorov–Smirnov test). **(E)** Gene Ontology analysis of transcripts with decreased TE upon *Eif2d* depletion (p-values adjusted for multiple tests using FDR). **(F)** Cumulative distributions of TPM of all expressed transcripts, transcripts with changed abundance upon *Eif2d* knock-down and transcripts with changed abundance upon *Denr* knock-down show an enrichment for highly abundant transcripts among *Eif2d*-responsive mRNAs (p-value calculated using Kolmogorov–Smirnov test). **(G)** Gene Ontology analysis of transcripts with increased abundance upon *Eif2d* depletion (p-values adjusted for multiple tests using FDR). **(H)** Gene Ontology analysis of transcripts with decreased abundance upon *Eif2d* depletion (p-values adjusted for multiple tests using FDR). **(I)** Cell proliferation analysis of NIH/3T3 cells transduced with con1 shRNA, con2 shRNA, *Denr* shRNA and *Eif2d* shRNAs shows that a larger fraction of *Eif2d* knock-down cells are found in the G1 phase of the cell cycle than is the case across control cells or *Denr*-depleted cells (p-values calculated using two-tailed unpaired *t*-test). **(J)** Normalised luminescence signals (firefly/Renilla) of *Klhdc8a* reporters after transduction in HeLa WT and *Eif2d* KO cells show no change in re-initiation rates upon *Eif2d* depletion (p-values calculated using two-tailed unpaired *t*-test).

A Alignment of mouse *Mcts1* and *Mcts2* coding sequences:

```
MmMcts1_CDS      ATGTTCAAGAAATTTGATGAAAAAGAAAATGTGTCCAACTGCATCCAGTTGAAAACCTCG      60
MmMcts2_CDS      ATGTTCAAGAAATTTGACGAGAAGGAAAGTGTGTCCAACTGCATCCAACTGAAAACTTCC      60
                 ***************** ** ** **** ******************  ******* **

MmMcts1_CDS      GTTATTAAGGGTATTAAAAATCAATTGCTAGAGCAATTTCCAGGTATTGAACCATGGCTT      120
MmMcts2_CDS      GTTATTAAGGGTATTAAGAGCCAACTGACTGAGCAGTTTCCAGGTATCGAGCCGTGGCTT      120
                 ***************** *  *** **   ***** *********** ** ** ******

MmMcts1_CDS      AATCAAATCATGCCTAAGAAAGACCCTGTGAAAATTGTCCGATGCCATGAACACATAGAA      180
MmMcts2_CDS      AATCAAATCATGCCTAAGAAAGATCCCGTCAAAATAGTGAGATGCCATGAACACATGGAA      180
                 *********************** ** ** ***** **  **************** ***

MmMcts1_CDS      ATCCTTACAGTAAATGGAGAATTACTGTTTTTTAGACAAAGAGAAGGGCCTTTTTATCCA      240
MmMcts2_CDS      ATCCTTACAGTCAACGGAGAATTACTGTTTTTCAGGCAGAGAAAAGGACCTTTTTATCCA      240
                 *********** ** ***************** ** ** *** **** ************

MmMcts1_CDS      ACTTTAAGATTACTTCATAAATATCCTTTTATCTTGCCACATCAGCAGGTTGATAAAGGA      300
MmMcts2_CDS      ACGCTAAGACTACTTCACAAATACCCGTTTATCCTGCCACACCAGCAGGTCGACAAAGGA      300
                 **  ***** ******* ***** ** ****** ******* ******** ** ******

MmMcts1_CDS      GCCATCAAATTTGTACTCAGTGGAGCAAATATCATGTGTCCTGGCTTAACTTCTCCCGGA      360
MmMcts2_CDS      GCCATCAAATTTGTGCTCAGTGGTGCAAATATCATGTGCCCGGGTTTAACGTCTCCTGGA      360
                 ************** ******** ************** ** ** ***** ***** ***

MmMcts1_CDS      GCTAAGCTTTATCCTGCTGCAGTAGATACTATTGTTGCAATCATGGCAGAAGGAAAACAA      420
MmMcts2_CDS      GCGAAGCTCTACACTGCTGCAGTAGATACCATCGTGGCGGTCATGGCAGAGGGGAAAGAG      420
                 ** ***** **  **************** ** ** **  ********** ** *** *

MmMcts1_CDS      CATGCTTTATGTGTGGGTGTCATGAAGATGTCTGCAGAAGATATTGAGAAAGTAAACAAA      480
MmMcts2_CDS      CATGCCCTGTGTGTCGGAGTCATGAAGATGGCTGCAGCAGACATTGAGAAAATCAACAAG      480
                 *****  * ***** ** ************ ****** *** ********* * *****

MmMcts1_CDS      GGAATTGGCATTGAAAATATCCATTATCTAAATGATGGTCTGTGGCATATGAAGACATAT      540
MmMcts2_CDS      GGGATCGGCATTGAGAATATCCATTATCTAAATGACGGGCTGTGGCACATGAAGACATAT      540
                 ** ** ******** ******************** ** ******** ************

MmMcts1_CDS      AAATGA      546
MmMcts2_CDS      AAGTGA      546
                 ** ***
```

B

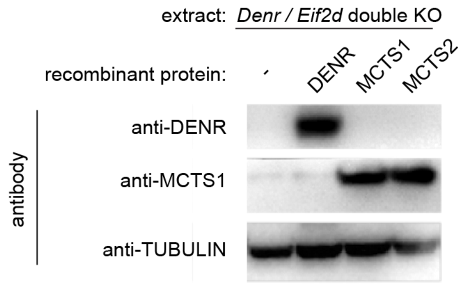

Appendix Figure S6. Comparison of mouse *Mcts1* and *Mcts2* coding sequences and antibody specificity. **(A)** Nucleotide alignment of *Mcts1* and *Mcts2* coding sequences from *Mus musculus*. **(B)** Immunoblot analysis of *Eif2d* and *Denr* double knockout HeLa *in vitro* translation extracts supplemented with the indicated recombinant proteins, using the same concentrations as in rescue experiments (0.5 µM). The anti-MCTS1 antibody is reactive against both MCTS1 and MCTS2.

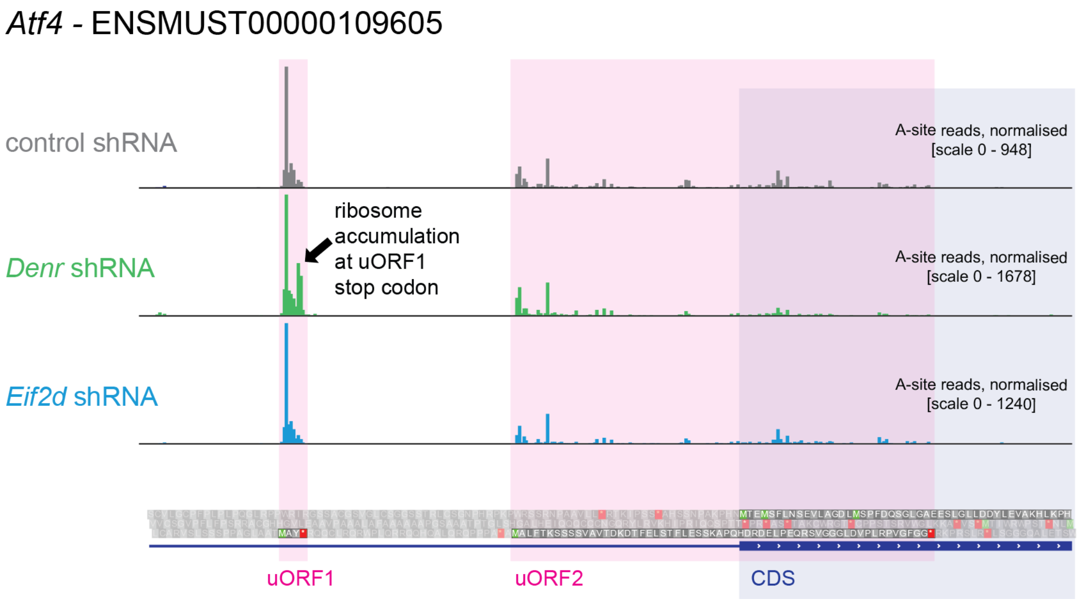

Appendix Figure S7. DENR depletion leads to an accumulation of ribosomes at the uORF stop codon on the endogenous *Atf4* transcript. Mapped footprint A-sites of mouse *Atf4* transcripts in control, *Eif2d* and *Denr* knock-down cells. Read numbers were normalised to library depth by subsampling and replicates were merged for increased coverage.

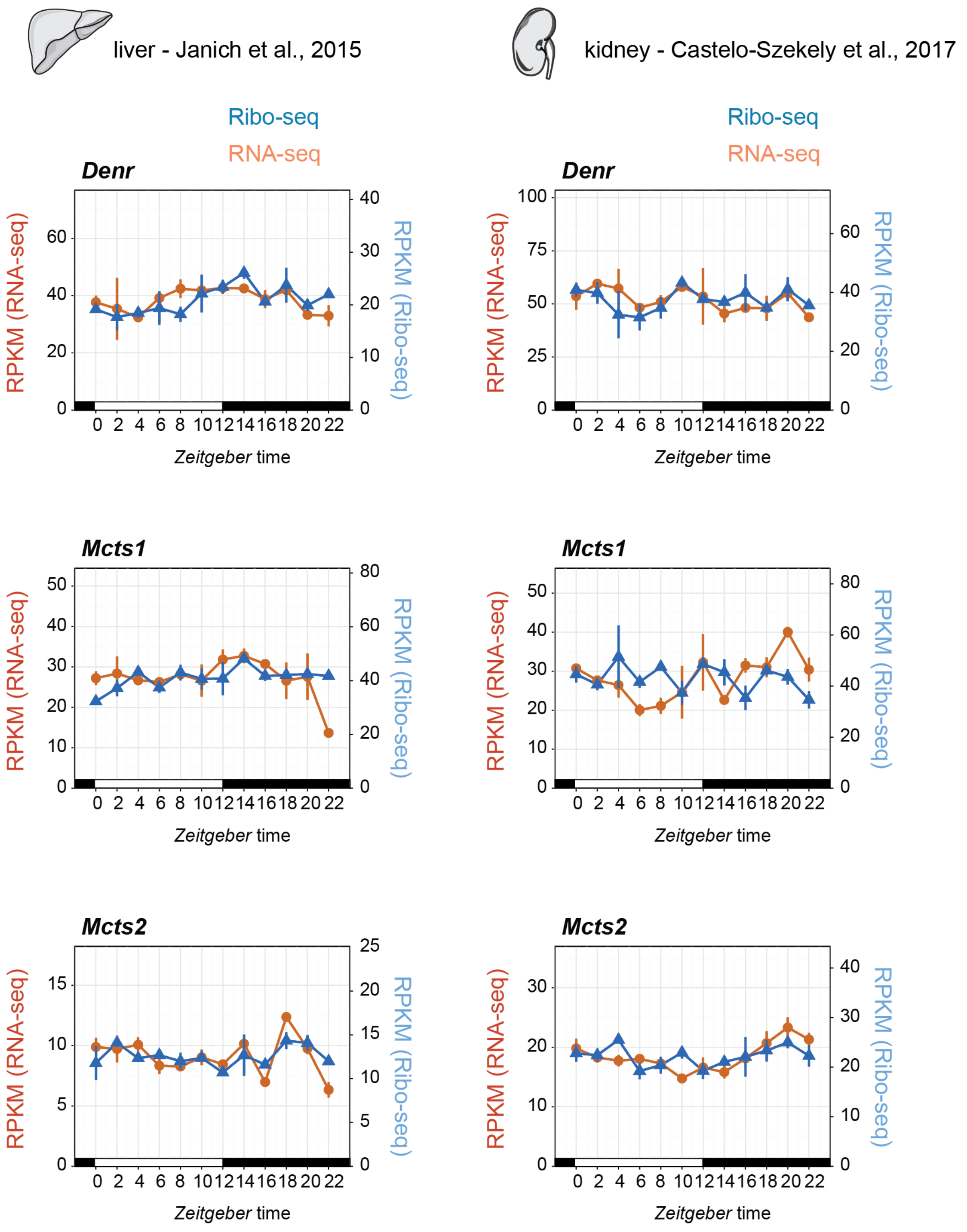

Appendix Figure S8. *Mcts2* expression in mouse liver and kidney. RPKM of *Denr*, *Mcts1* and *Mcts2* around-the-clock in mouse liver and kidney confirms expression of *Mcts2* in adult mice. Liver and kidney RNA-seq and Ribo-seq data were taken from previously published studies (Castelo-Szekely *et al.*, 2017; Janich *et al.*, 2015).
