# Supplementary figures and images for: MCTS2 and distinct eIF2D roles in uORF-dependent translation regulation revealed by in vitro re-initiation assays
Source: EMBO J. 2025 Jan 2;44(3):854–76. doi: 10.1038/s44318-024-00347-3 (PMC11790910; doi:10.1038/s44318-024-00347-3)

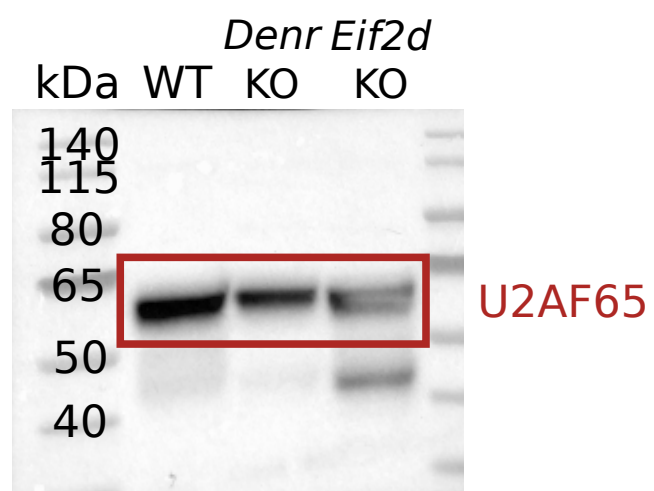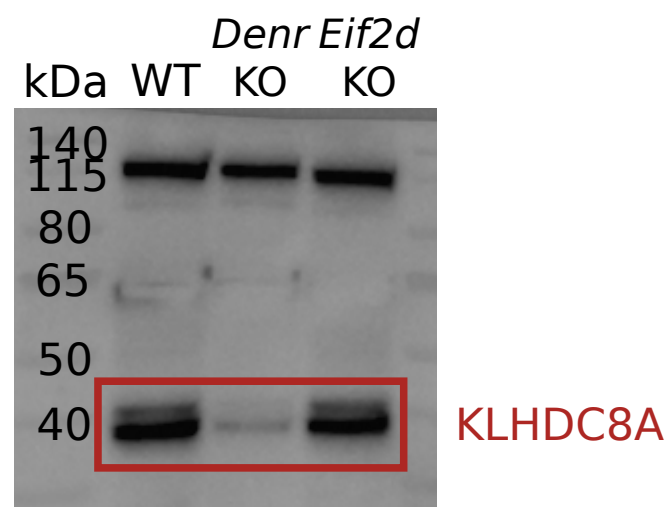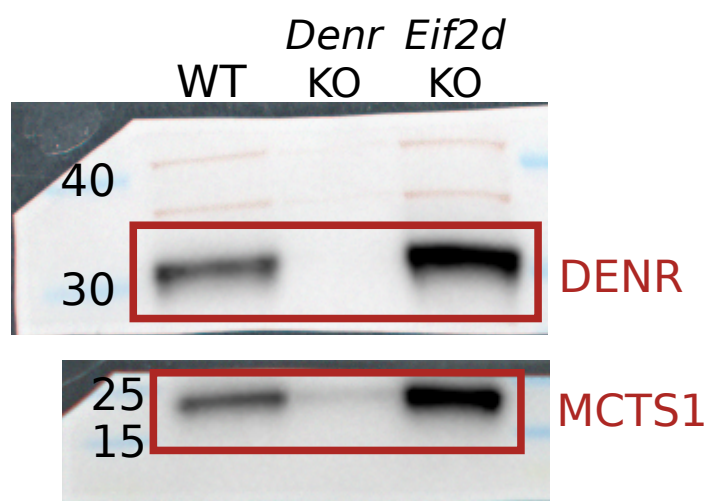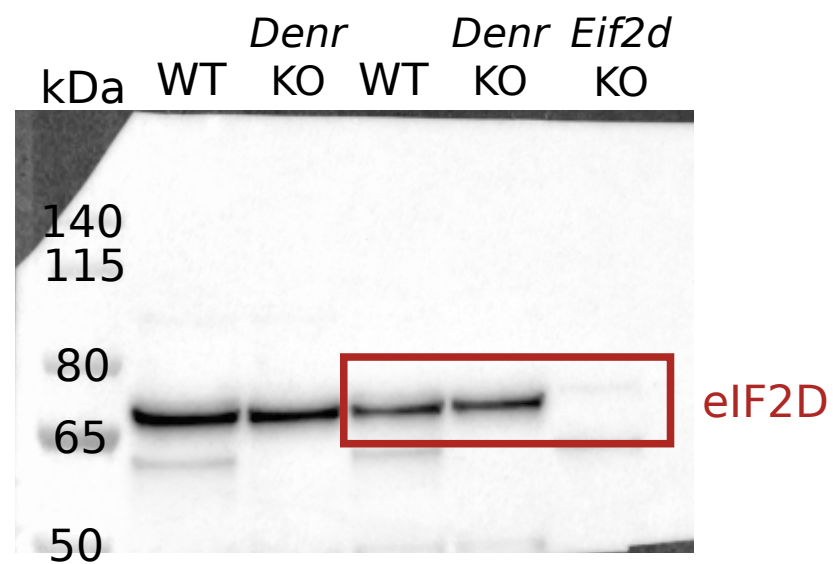

Supplement: Supplementary file 5 — Source data Fig. 1 [file 44318_2024_347_MOESM5_ESM.zip › SD Figure 1/1H/WB_HeLa_KLHDC8A.pdf]

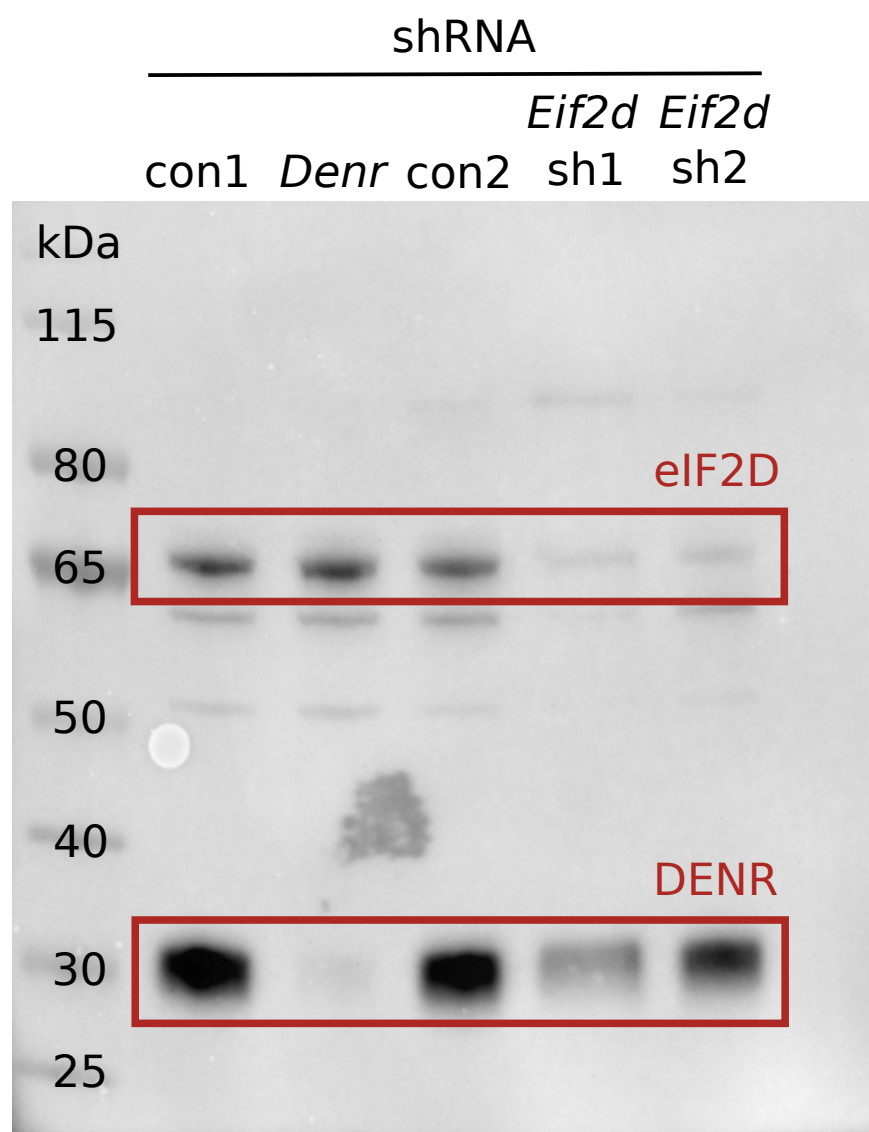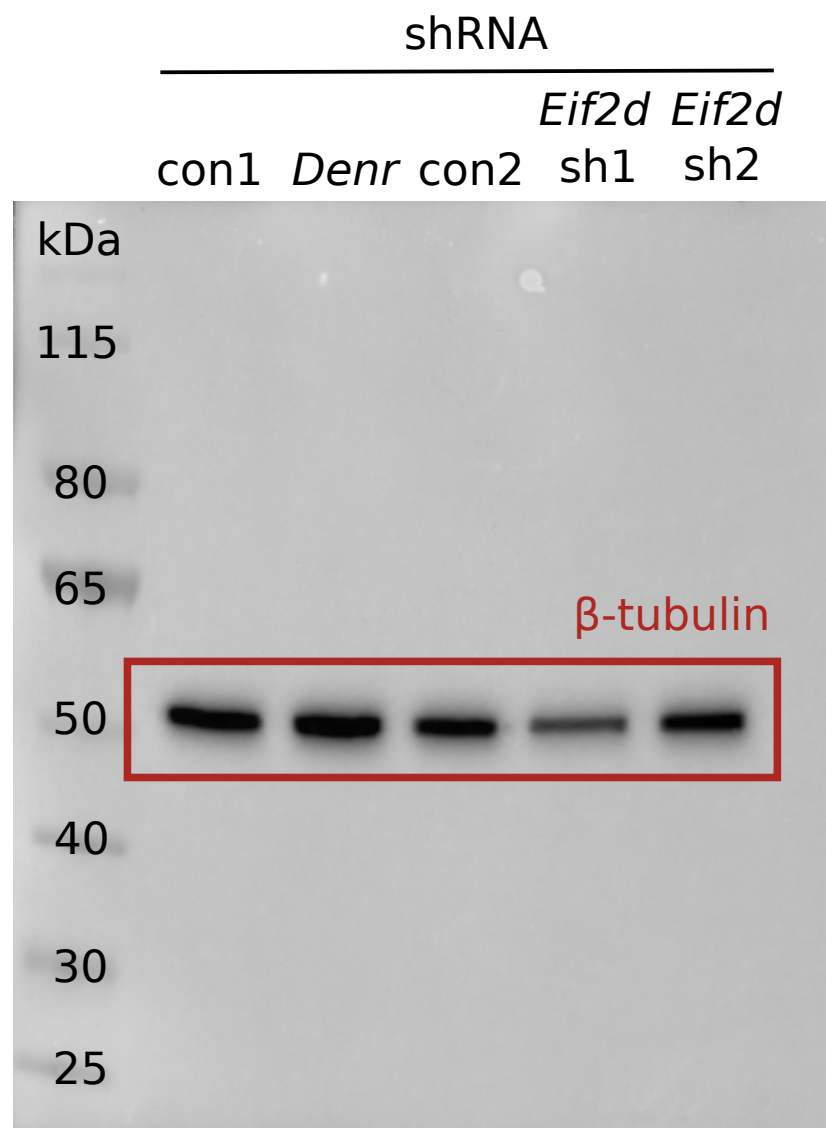

Supplement: Supplementary file 5 — Source data Fig. 1 [file 44318_2024_347_MOESM5_ESM.zip › SD Figure 1/1A/WB_shRNAs.pdf]

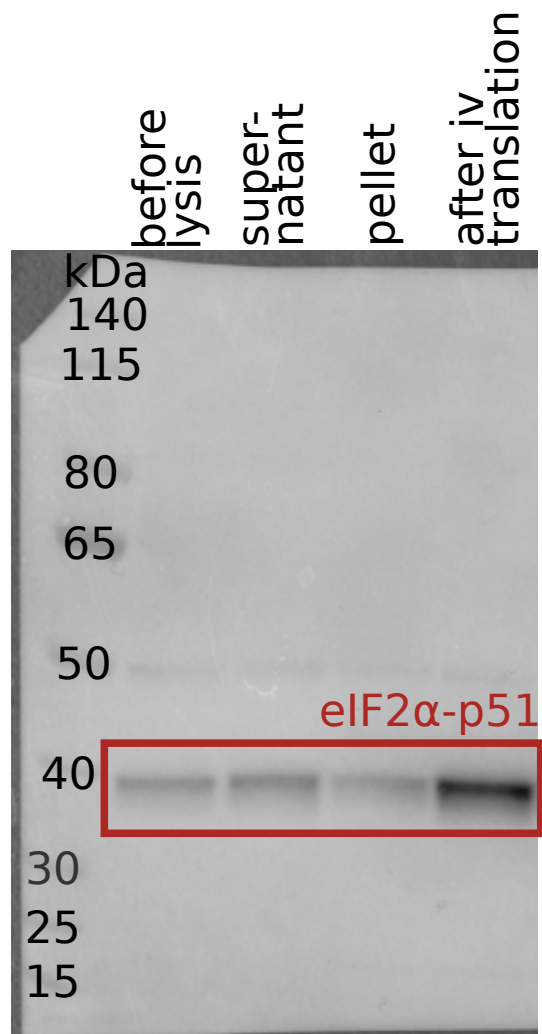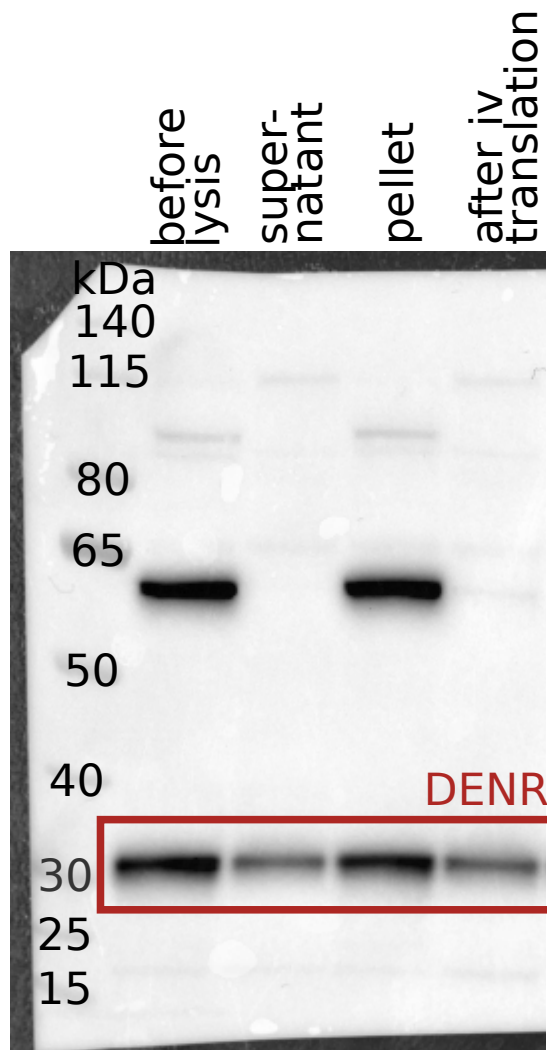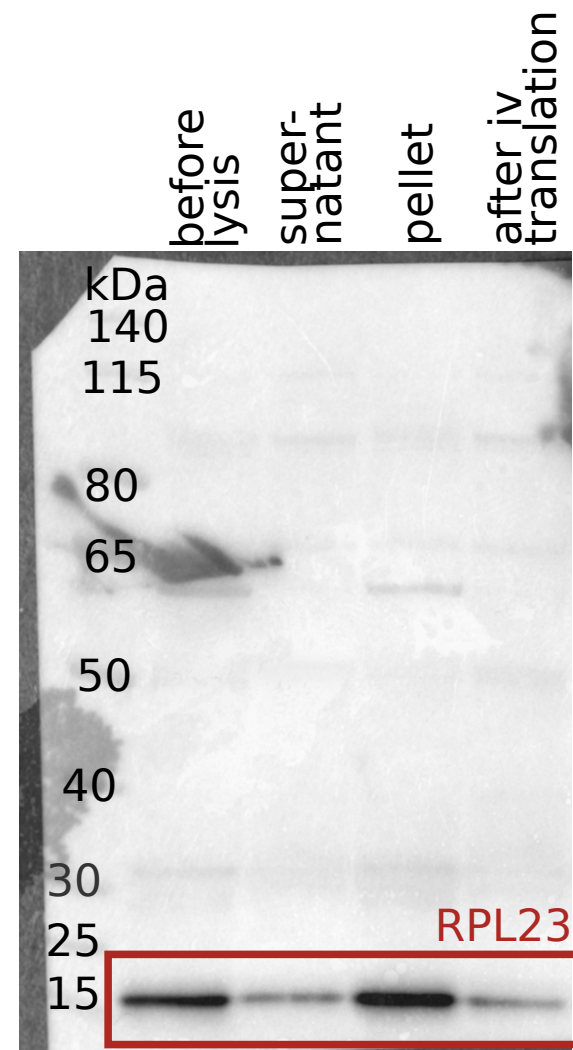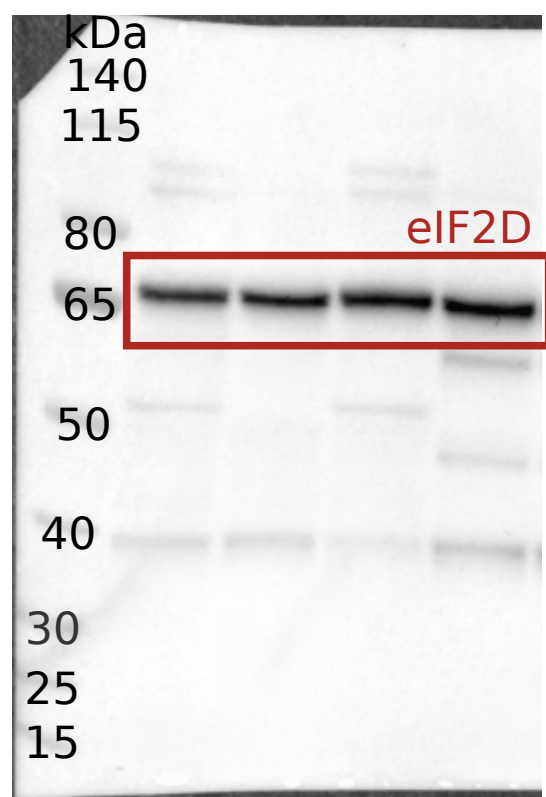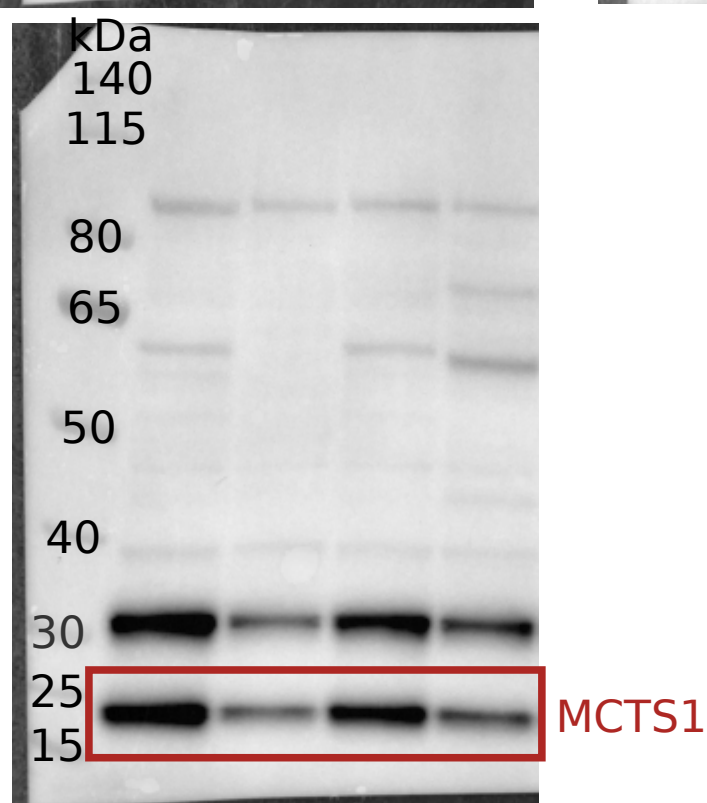

Supplement: Supplementary file 6 — Source data Fig. 2 [file 44318_2024_347_MOESM6_ESM.zip › SD Figure 2/2B/WB_HeLa_lysate.pdf]

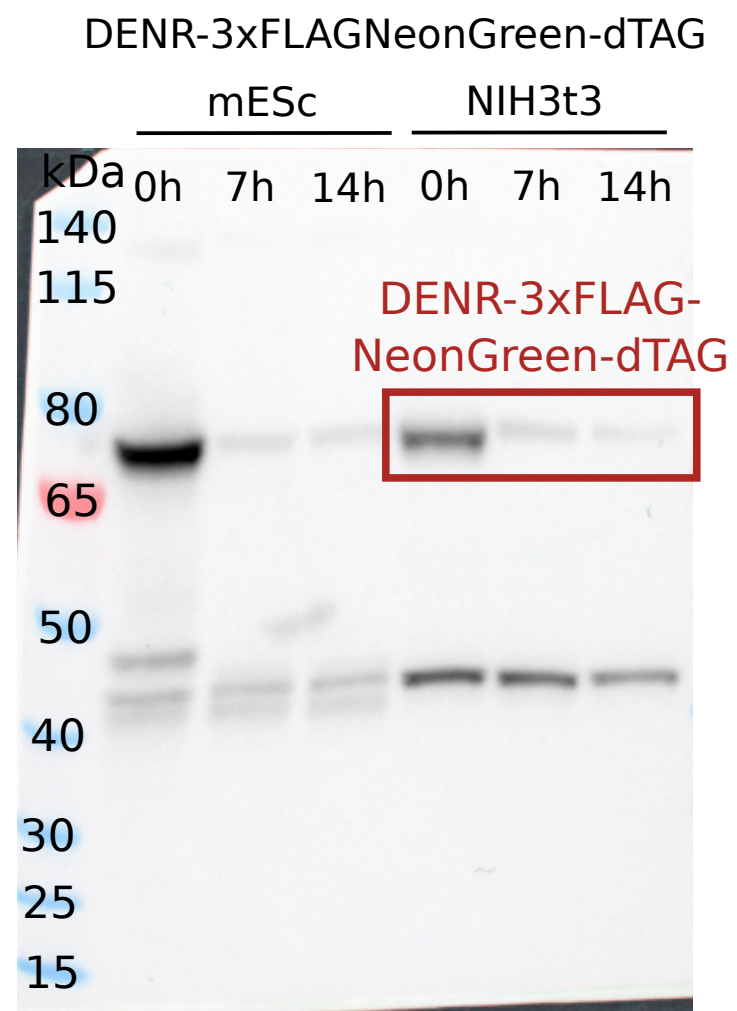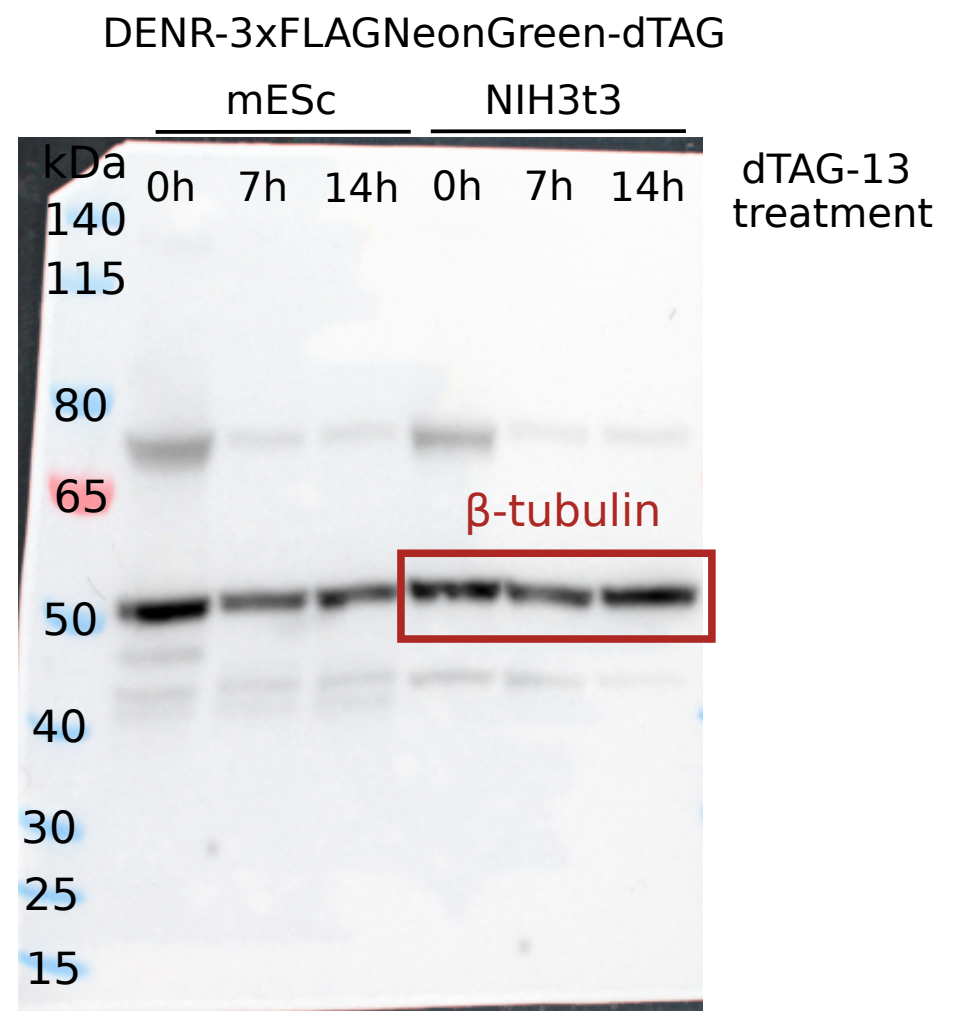

Supplement: Supplementary file 9 — Source data Fig. 5 [file 44318_2024_347_MOESM9_ESM.zip › SD Figure 5/5C/WB_dTAG_treatment.pdf]

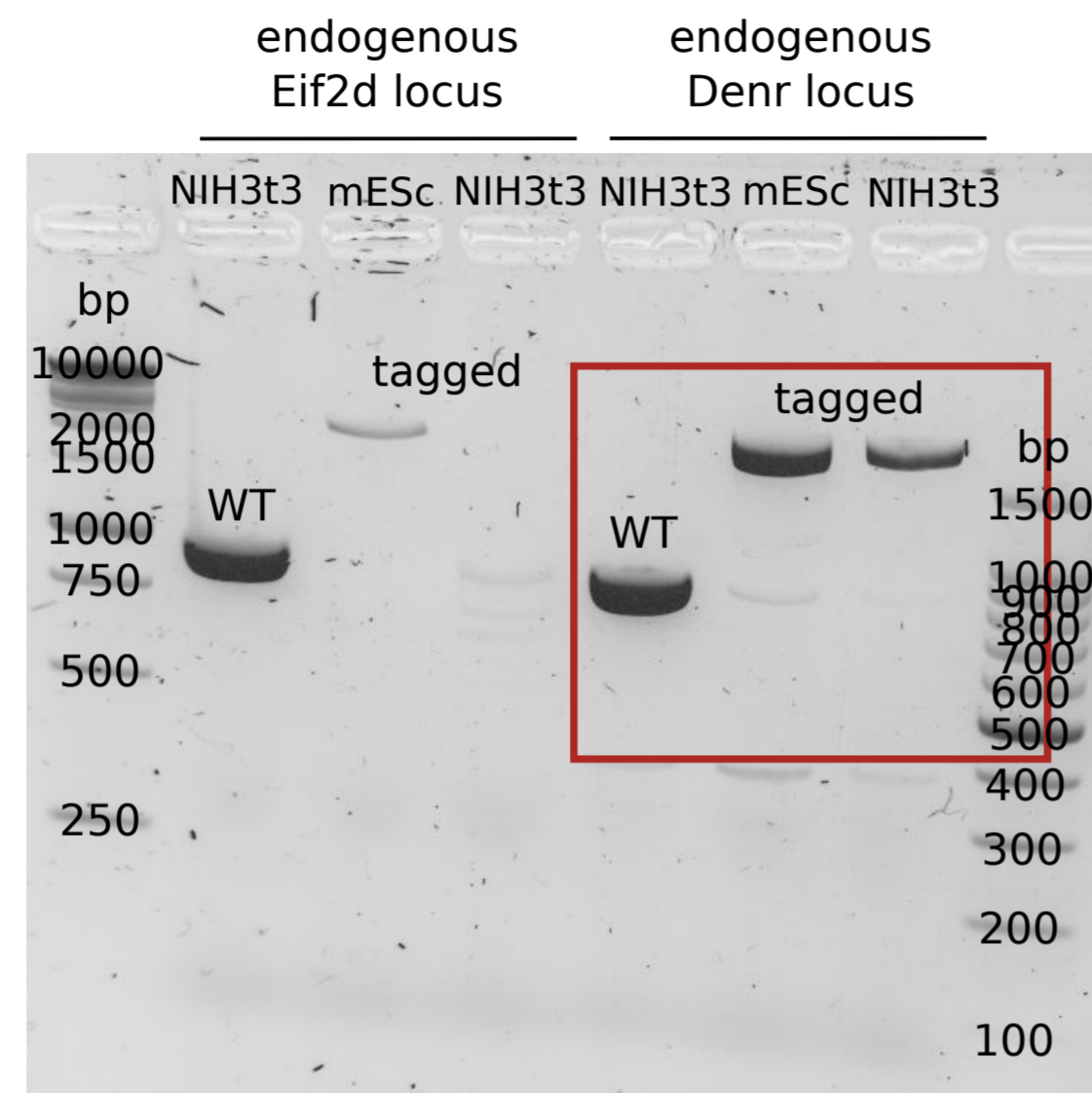

Supplement: Supplementary file 9 — Source data Fig. 5 [file 44318_2024_347_MOESM9_ESM.zip › SD Figure 5/5B/Genotyping_PCR_Denr-tagged.pdf]
