# Supplementary material for: MCTS2 and distinct eIF2D roles in uORF-dependent translation regulation revealed by in vitro re-initiation assays
Source: EMBO J. 2025 Jan 2;44(3):854–76. doi: 10.1038/s44318-024-00347-3 (PMC11790910; doi:10.1038/s44318-024-00347-3)
Supplement: Supplementary file 6 — Source data Fig. 2 [file 44318_2024_347_MOESM6_ESM.zip › SD Figure 2/2C/WB_GADD34_vs._noGADD34.pdf]

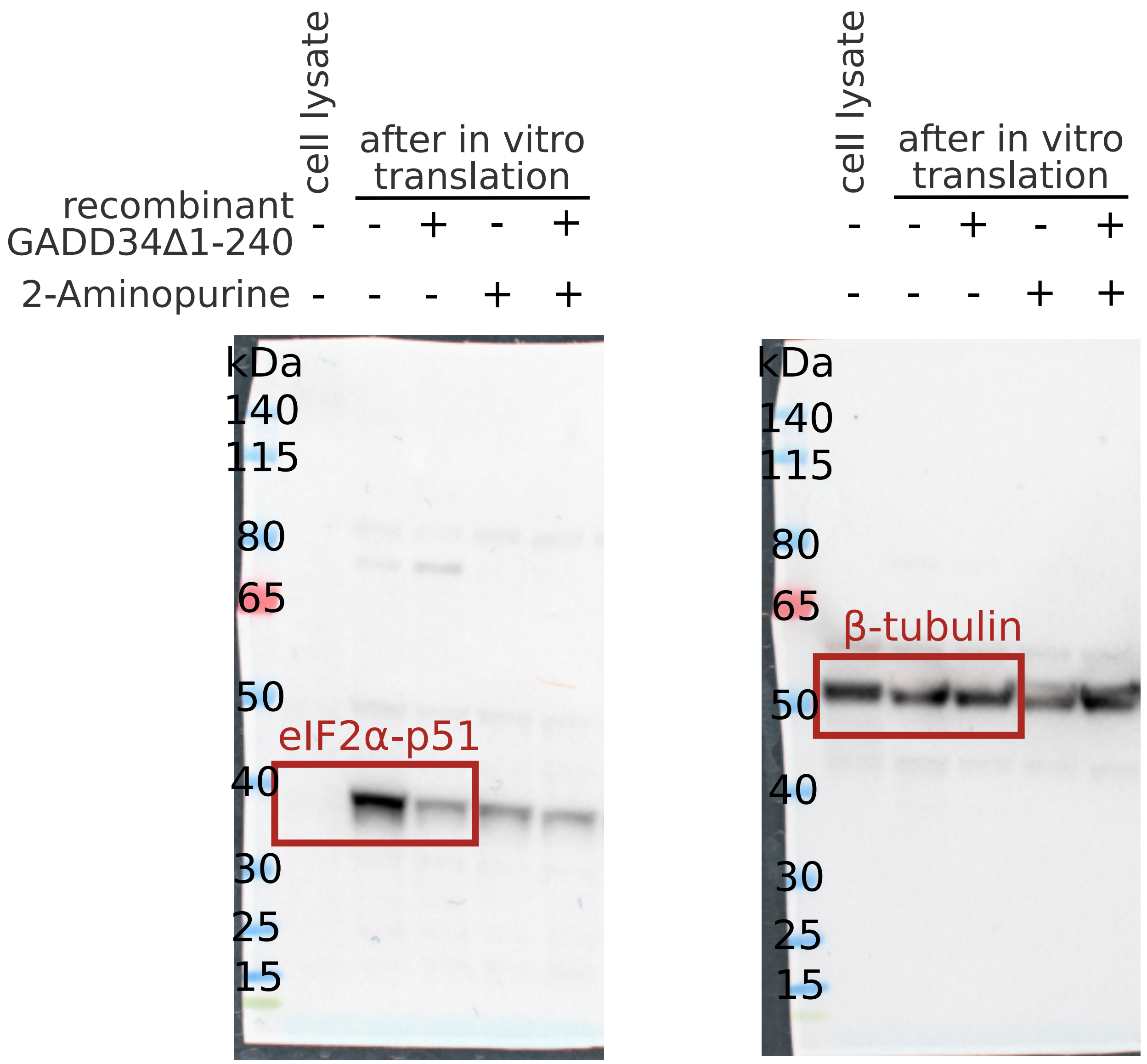

cell lysate
after in vitro translation
recombinant GADD34Δ1-240
2-Aminopurine
kDa
140
115
80
65
50
40
30
25
15
eIF2α-p51
β-tubulin
